# Biomimetic on-chip filtration enabled by direct micro-3D printing on membrane

**DOI:** 10.1038/s41598-022-11738-z

**Published:** 2022-05-17

**Authors:** Hongxia Li, Aikifa Raza, Shaojun Yuan, Faisal AlMarzooqi, Nicholas X. Fang, TieJun Zhang

**Affiliations:** 1 Department of Mechanical Engineering, Masdar Institute, Khalifa University of Science and Technology, P.O. Box 127788, Abu Dhabi, UAE; 2 College of Chemical Engineering, Sichuan University, Chengdu, 610065 China; 3 Department of Chemical Engineering, Masdar Institute, Khalifa University of Science and Technology, P.O. Box 127788, Abu Dhabi, UAE; 4 Department of Mechanical Engineering, Massachusetts Institute of Technology, 77 Massachusetts Avenue, Cambridge, MA 02139 USA

**Keywords:** Fluidics, Mechanical engineering

## Abstract

Membrane-on-chip is of growing interest in a wide variety of high-throughput environmental and water research. Advances in membrane technology continuously provide novel materials and multi-functional structures. Yet, the incorporation of membrane into microfluidic devices remains challenging, thus limiting its versatile utilization. Herein, via micro-stereolithography 3D printing, we propose and fabricate a “fish gill” structure-integrated on-chip membrane device, which has the self-sealing attribute at structure-membrane interface without extra assembling. As a demonstration, metallic micromesh and polymeric membrane can also be easily embedded in 3D printed on-chip device to achieve anti-fouling and anti-clogging functionality for wastewater filtration. As evidenced from *in-situ* visualization of structure-fluid-foulant interactions during filtration process, the proposed approach successfully adopts the fish feeding mechanism, being able to “ricochet” foulant particles or droplets through hydrodynamic manipulation. When benchmarked with two common wastewater treatment scenarios, such as plastic micro-particles and emulsified oil droplets, our biomimetic filtration devices exhibit 2 ~ 3 times longer durability for high-flux filtration than devices with commercial membrane. This proposed 3D printing-on-membrane approach, elegantly bridging the fields of microfluidics and membrane science, is instrumental to many other applications in energy, sensing, analytical chemistry and biomedical engineering.

## Introduction

Membrane filtration and separation has been widely utilized in bio-medical, water and environment applications^1–6^. In the broad water purification and wastewater filtration process, purified water permeates through the membrane, whereas the contaminants like plastic micro-particles, oil droplets and solutes are rejected by the membrane. In spite of well-recognized advantages of membrane filtration (i.e. high-quality permeates, low space usage, easy automation and control), membrane fouling and clogging remains a major bottleneck in effective water filtration^7–10^. The integration of mass transport control by means of filtration membrane into microfluidic devices has shown substantial growth for high throughput development of anti-fouling/clogging solutions^11–15^.

**Figure 1:**
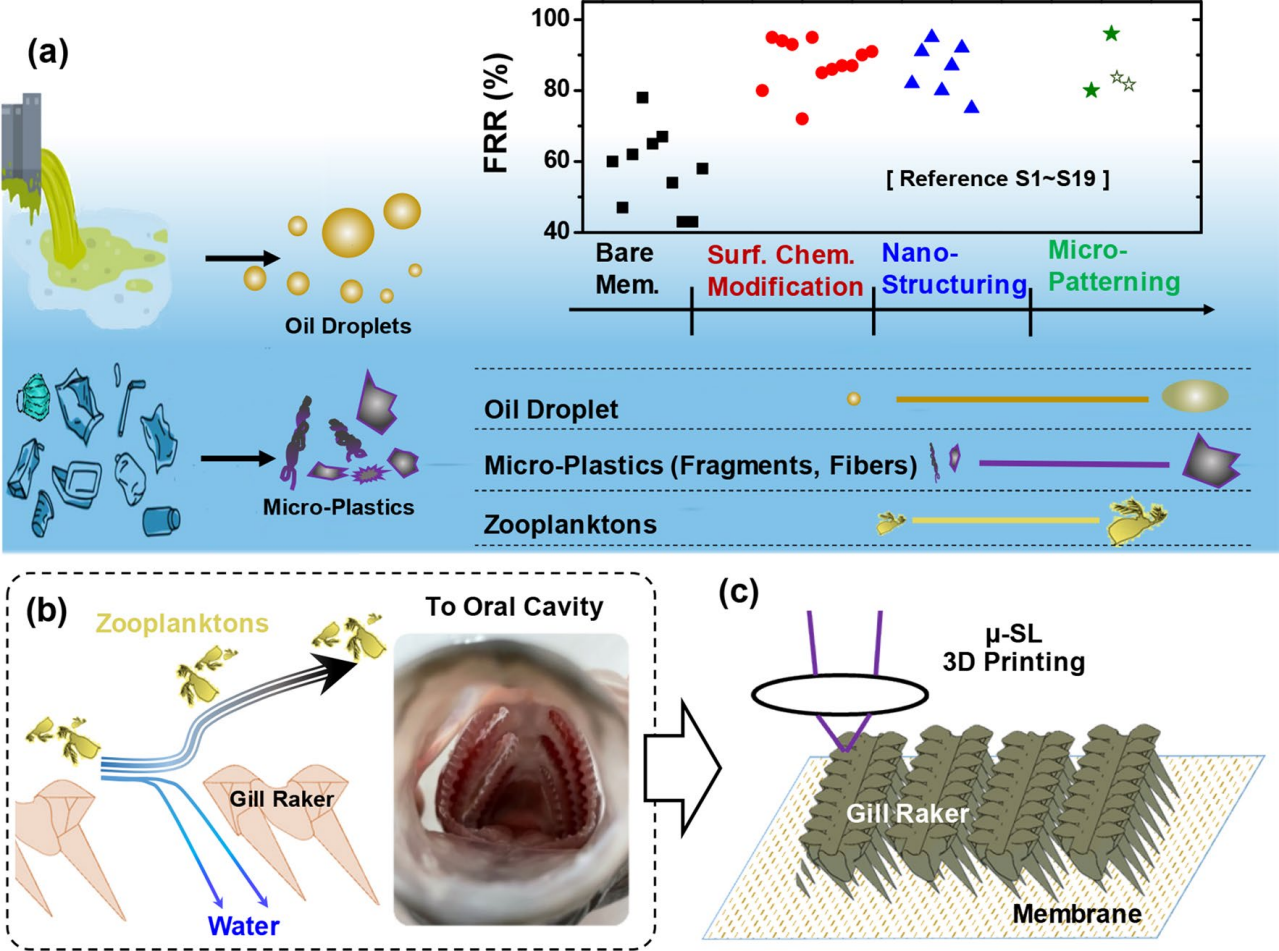
Bio-inspired anti-fouling membrane filtration device enabled by 3D printing-on-membrane. (**a**) Membrane filtration for wastewater treatment. The flux recovery ratio (FRR) for existing anti-fouling/clogging strategies are summarized from reported literatures (also see Table S1 of Supporting Information), including surface chemistry modification, nano-structuring, and micro-patterning. The sizes of oil droplet in produced water, micro-plastics found in marine environment, and food particles from fish gut content analysis. (**b**) Illustration of fish filtering food particles out of water via hydrodynamic manipulation and the optical image of fish mouth with gill raker structures. (**c**) Fish-mimicked anti-fouling membrane filtration device enabled by 3D printing on membrane. The gill-shaped structures are directly printed on membrane surface for anti-fouling filtration by using micro-stereolithography 3D printing system.

Currently, anti-fouling/clogging strategies are mainly focused on novel membrane material development^16,17^ and membrane surface modification^9,18–20^. Membrane surface chemistry and wettability, highly affects surface-foulant interaction and fouling tendency: membrane surface with super-hydrophobicity and underwater oleophobicity is desired to mitigate foulant adhesion^17,21,22^. Intensive research efforts have demonstrated that surface coating of metal oxide^9^ and even photocatalytic materials^23,24^ can make the membrane exhibit superior anti-fouling capability toward organic fouling repellency and degradation. Such chemical approaches have been widespread for its easy implementation and high flux recovery ratio (FRR) (see Fig. 1a), however, besides coating adhesion/degradation issues, environmental concerns often arise with chemical waste disposal. As an alternative, chemical-free anti-fouling/clogging strategy becomes highly attractive. Surface patterning, creating topological structures on membrane surfaces, can manipulate the local hydrodynamics and the corresponding foulant-surface interaction^25–30^. With properly designed surface structures, the flow field near the membrane surface can be controlled to inhibit the deposition and accumulation of foulants particularly micro-sized foulant particles or droplets. These membrane structures have a comparable size with oil droplets in produced water^31^, and harmful micro-plastic fragments or fibers found from fish gut content analysis^32^ (see Fig. 1a). Moreover, by regulating the local velocity field, the shear stress induced at the foulant-membrane interface can further enable the detachment and removal of the foulants^28,33^.

In membrane surface patterning, structure geometry is the key. Different surface structures like grooves and pyramids have demonstrated certain anti-fouling properties^27,33^. Besides, aquatic creatures after evolution have exploited the principle of growing surfaces with near-optimum structures, such as fish scales and gill rakers, sea sponge lattices and ridges with dimensions ranging from millimeters down to several nanometers. In particular, the fish feeding process, filtering plankton and other food particles out of the water (see Fig. 1b), have inspired many laboratory attempts to mitigate membrane fouling^34–37^. Often, these biological structures exhibit remarkable complexity, and replicating them is becoming feasible with the aid of emerging 3D printing technique^38^. Recent advances in micro-stereolithography (µ-SL) 3D printing have made possible to fabricate complex structures with as small as 2 µm feature size^39^, however, it is still challenging to integrate micro-/millimeter surface architectures with membranes of nanometer pores in one step^40–42^. Due to the significant feature size difference and printing resolution limitations, the nanoporous membrane has to be manufactured separately. Extra assembling is always required, but it is simply impossible to assemble a large amount of 3D-printed discrete structural elements (i.e., spikes, gill rakers) with a membrane sheet. Direct printing on membrane promises great potential in efficiently integrating the membrane and complex surface structures as an all-in-one device. To the best of our knowledge, such an assembly-free additive device fabrication approach has not been reported yet for filtration purpose.

In this work, we propose a novel micro-3D printing-on-membrane approach. Its unique advantage is demonstrated by directly printing fish gill-shaped structures on porous membrane to mimic fish feeding mechanism for anti-fouling/clogging filtration (see Fig. 1c). The anti-fouling/clogging performance of as-fabricated devices is assessed by the filtration of wastewater containing plastic micro-particles and surfactant-stabilized emulsion, one of the most challenging problems in oily water treatment. With the proposed printing-on-membrane approach, we also incorporate metallic micro-mesh with polymeric membrane to make hybrid-material filtration devices. In-situ flow visualization is performed to seek deep insight of antifouling/clogging mechanism of as-fabricated microfluidic filtration devices.

## Experimental methods

### Materials

For lab experiments, the oil-in-water emulsion is prepared by adding 10 mL corn oil (Afia, local market) and 1 g sodium dodecyl sulfate (SDS, Sigma Aldrich) into 100 mL water. The solution is stirred for 1 h under 1000 rpm speed. For micro-plastic suspension, 2 g of the commercially-available polyethylene microspheres (Cospheric, 1.10 g/cc 10–90 µm) are mixed with 100 mL water. SDS is also added to enhance the suspension’s uniformity. After mixing, the suspension is stirred for 8 h, further breaking the plastic microspheres into smaller irregular-shaped particles. All the materials are used without any further purification.

### 3D printing-on-membrane procedures

Our proposed printing-on-membrane technique for creating a 3D-structured filtration membrane is illustrated in Fig. S1. A µ-SL printing system (BMF, S130) is used to fabricate the 3D structures through layer-by-layer photo-polymerization^43,44^. At Step 1, the membrane (Whatman, Nytran N, ~ 200 nm) is submerged with the printing ink (BMF, S130 HDDA-based Ink) for 1 h, allowing all the pores to be filled with ink. Then, the ink-saturated membrane is placed on the printing stage or on the previously-printed layer, as shown in Step 2. In our experiments, the membrane thickness is around 140 µm. So we also keep the liquid gap with the same distance when printing the membrane-embedded layer. With the intrinsic layer-by-layer printing process of the µ-SL 3D printing system, the newly printed layer is then formed with embedded membrane. Note that the printing time of this membrane-embedded layer is reasonably prolonged depending on the thickness and porosity of membrane material, to allow the ink sufficiently being cured both inside the pores and underneath the membrane.

## Results

### Fish-mimicked on-chip membrane filtration device

**Figure 2:**
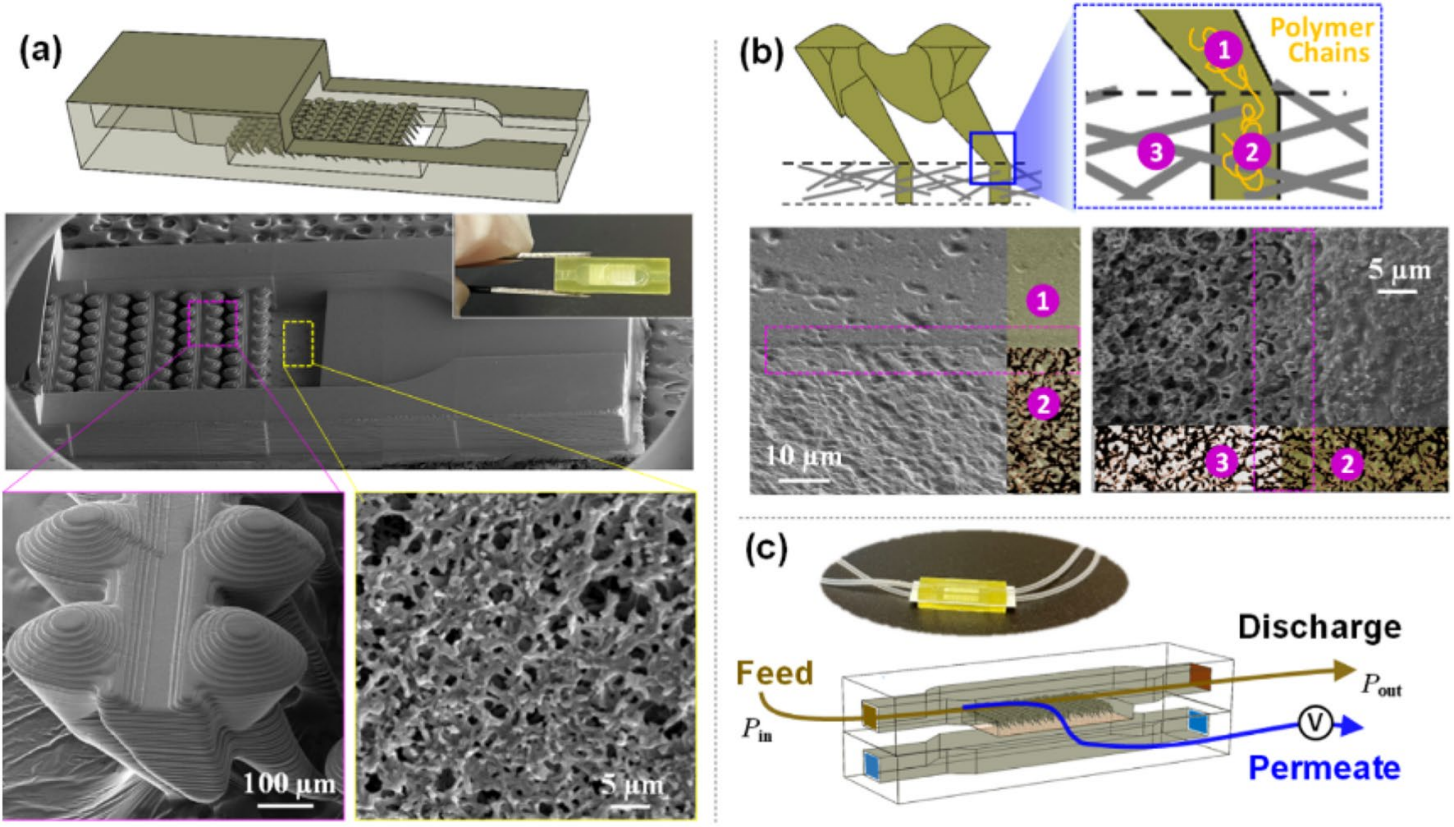
3D-printed on-chip membrane filtration device with self-sealed interfaces. (**a**) Schematic and SEM images of 3D-printed fish-mimicked membrane filter. Membrane is embedded as printing substrate, where the fish gill structures and supportive frames are directly printed on it. **(b)** Self-sealed interfacial region between 3D-printed structure and membrane. When directly printing on membrane, the photo-polymerization and cross-linking process is illustrated in the schematic diagram, and the SEM images show the interfacial regions of printed structures and membrane (highlighted the dash box). The solidified printing resin within the membrane pores strongly bonds the printed structures above membrane, spontaneously achieving self-sealing properties. (**c**) Schematic of microfluidic platform for high-throughput filtration performance evaluation. Optical image of as-fabricated device is also provided. The microfluidic chip size is 7 mm in width and 19 mm in length.

The fish-mimicked membrane is fabricated by directly 3D printing gill raker-shaped structures on membrane surface. In the printing, the membrane (Whatman, Nytran N, ~ 200 nm) was used as the substrate. By sequentially projecting the stack of images sliced from a 3D gill raker model, the overlaying surface structures was then printed on membrane. Here, we also designed the fish-mimicked membrane into a microfluidic filtration device. The device integrates all the functional components, including the fish gill-shaped structures, inlet and outlet opening, and supportive frames embedded with porous membrane. With the same printing procedure, we are able to create an all-in-one microfluidic filtration device. The scanning electron microscopy (SEM) image in Fig. 2a shows successful mimicking of gill raker-shaped structures on membrane surface in a microfluidic filtration device. The cross-sectional views are given in Fig. S2a.

Successful fabrication of the fish-mimicked membrane demonstrated the great capability of micro-3D printing in integrating existing materials, not limited to membrane, with newly-printed structures (i.e. supportive frames or gill structures). More importantly, the directly-printed structures have excellent bonding with the membrane, because the polymer chains are cross-linked into membrane pores during the photo-curing process. This is evidenced from the cross-section view under the SEM in Fig. 2b, showing the internal morphology of porous membrane after 3D printing. The membrane pores under the printed structure are filled with solidified resin completely, while no void space is observed at the structure-membrane interfacial regions (see the highlighted dash boxes in Fig. 2b). In another words, direct 3D printing on membrane is able to achieve strong bonding of as-printed structures on membrane and self-sealing properties without extra assembling.

The as-fabricated filtration device can be directly used in a desktop filtration platform (see Fig. S3). The optical images of as-printed microfluidic filtration devices after tubing are shown in Fig. 2**c**. The operation conditions of a crossflow filtration process can be controlled by the injection pressure at the inlet and back pressure at the outlet, while the permeate flux is monitored with a flow rate sensor. From the flux decline, we are able to evaluate the anti-fouling/clogging performance of 3D-structured membrane. Such “print-and-play” microfluidic filtration device enables a quick, high-throughput development of novel functional membrane.

### Filtration performance evaluation with micro-plastics and emulsion

**Figure 3:**
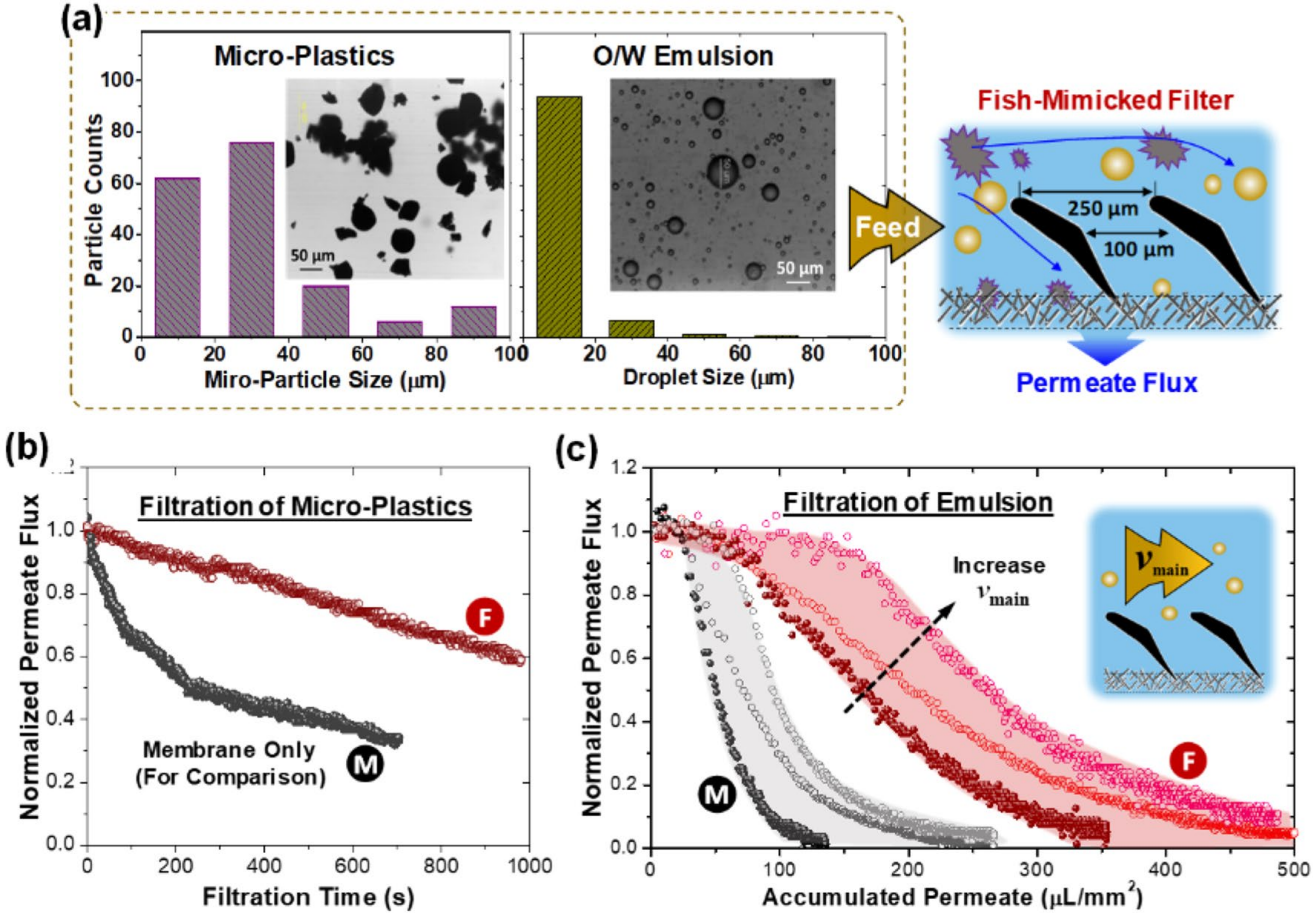
Anti-fouling performance evaluation with two filtration benchmark cases: surfactant-stabilized emulsion and plastic micro-particle/water mixture. (**a**) The particle/droplet size distribution and inset optical image of wastewater containing micro-plastic particles (left) and emulsified oil droplet (right). (**b,c**) Filtration durability when filtering micro-plastic particles and emulsion. In comparison of using the bare membrane (black curve), the decay of permeate flux is mitigated significantly with the fish-mimicked filter (red curve). By increasing the main flow velocity, the durability is further prolonged as plotted in (**c**).

As the filtration benchmark for as-fabricated membrane, we chose two of the most challenging wastewater treatment problems: surfactant-stabilized emulsion and plastic micro-particles. For lab experiments, we have prepared oil-in-water emulsion and plastic micro-particles as aquatic suspension (see Materials). Their morphologies and particle/droplet size distributions are given in Fig. 3a. The anti-fouling/clogging performance of as-fabricated devices was evaluated by permeate flux durability. For comparison, we also tested the bare membrane without any surface structure as a reference. During the filtration, the values of inlet and outlet pressure are maintained as 80 and 40 mbar, respectively. As expected, the normalized permeate flux gradually decreases for both membranes when filtering the plastic micro-particles (Fig. 3b). When using the bare membrane, the permeate flux declines severely to 40% of its initial flux within 10 min operation. Surprisingly, the fish gill structured membrane can maintain as high as 80% of its original performance. Being able to maintain a high permeate flux with a long filtration period indicates the effectiveness of the surface structures in mitigating particle deposition on membrane for anti-fouling/clogging filtration. Note that the sizes of plastic micro-particles range from 10 to 90 µm, much smaller than the gap between two adjacent gill elements. By using the oily wastewater, which contains even smaller emulsified oil droplets (majority ~ 20 µm), the fish-mimicked filter also shows better durability than using the bare membrane (see Fig. 3b, the solid circles). The findings indicate that the function of gill structures is more complex than simple sieving. In fact, it is exactly our original purpose of printing the fish gill structures on membrane, mimicking the hydrodynamic mechanism of aquatic animals filtering plankton and other food particles out of the water^36^. The role of hydrodynamics on anti-fouling is also evidenced from the influence of main flow velocity on filtration durability. In the experiments, we increased the main flow velocity by changing the injection pressure from 80 to 100 and 120 mbar, respectively. The values of normalized permeate flux are plotted in Fig. 3c with empty circles. To have a fairer comparison, we used the accumulated permeate rather than filtration time as the x axis when plotting the permeate flux. When the accumulated permeate reaches 200 µL/mm^2^, the bare membrane has been completely blocked with a near zero permeate flux, while the fish-mimicked membrane is able to sustain 38% of its initial flux. By further increasing the main flow velocity, the decline of permeate flux is further mitigated, maintaining as high as 80%. The durability of permeate flux is significantly prolonged with the increased main flow velocity.

### Hydrodynamic anti-clogging mechanism from “Ricochet”

**Figure 4:**
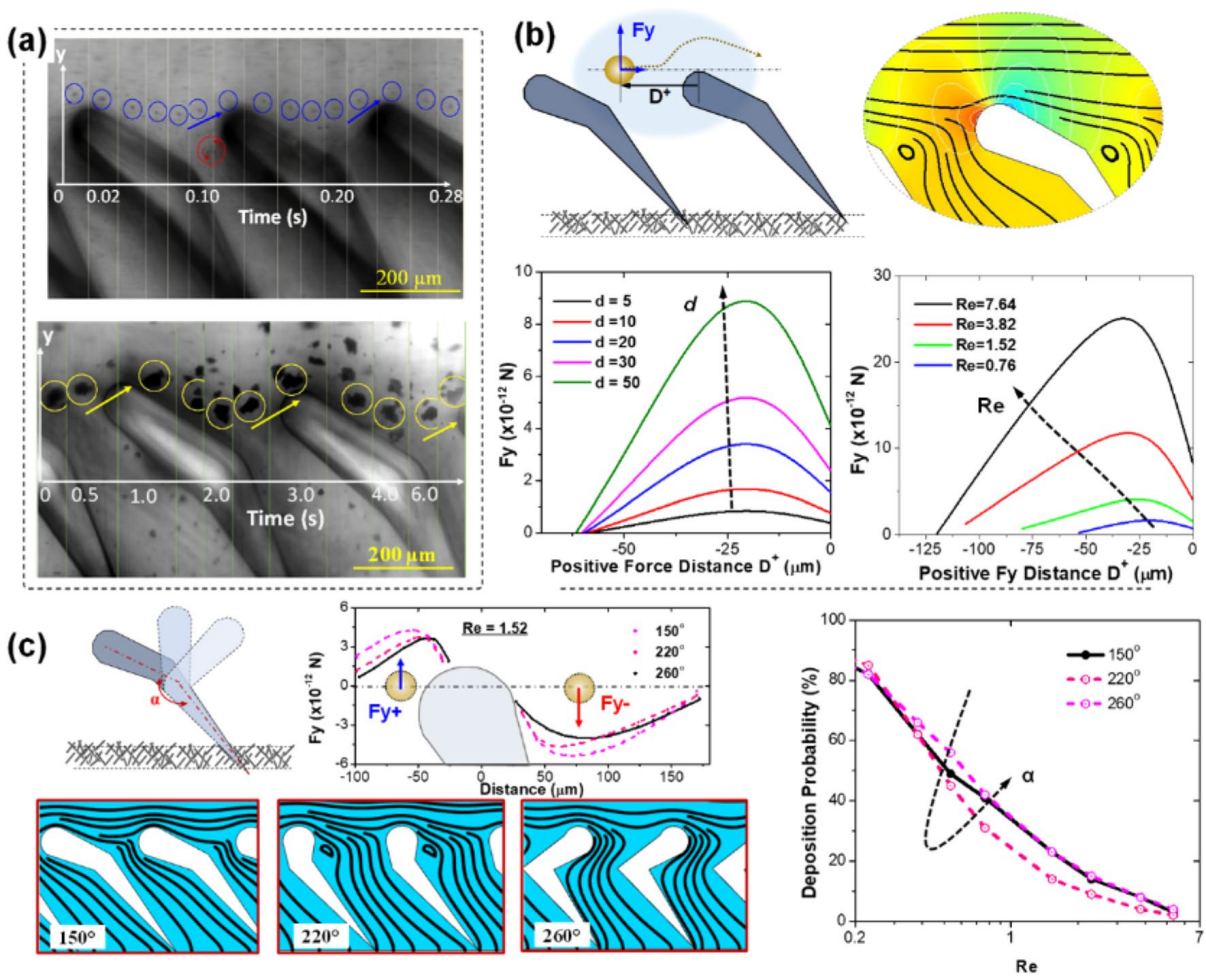
Ricochet effect induced by the fish gill-mimicked structures on membrane surface. (**a**) Optical snapshots showing the trajectory of oil droplets (top) and plastic micro-particles (bottom) when flowing above the fish gill-shaped structures on membrane surface. (**b**) Schematic of the droplet in the main flow (left) and numerical simulation results (right) showing the streamlines (black curves) and pressure distribution (rainbow color indicating high to low values) in the flow field. The curves show the influence of droplet size and Reynolds number on the lift force F_y_ ^+^ exerting on droplet. (**c**) The influence of gill shapes on the flow patterns, forces and oil droplet deposition probability.

The extraordinary anti-fouling/clogging performance of fish gill-structured membrane originates from the unique flow behavior of foulant droplet/particle during the filtration process. In fact, enabling an in-situ flow imaging is another merit of our proposed microfluidic membrane devices. Figure 4a shows the flow trajectories of oil droplets and plastic micro-particles under the optical microscope when passing above the gill-shaped structures. Take the oil droplet in the first row of Fig. 4a as an example. This figure is a combination of 16 consecutive images captured every 0.02 s. From its trajectory, we find that as the droplet approaches a gill element, it is entrained by the permeate flow to the gap (see t = 0.02 to 0.08 s the “blue circles”). However, the droplet gets abruptly diverted away from the gap due to the vortices (highlighted in red circle, t = 0.10 s) and encounters the leading edge of the next gill element, where the droplet ricochets away from the gap and return to the main stream (t = 0.10 to 0.12 s). This process repeats at the next gill element (t = 0.14 to 0.24 s) and causes the droplet excluded from the permeate. In this way, even though the droplet size is much smaller than the gaps between two neighboring gill elements, it does not enter the gap but retains in the main flow. Similar trajectory of plastic micro-particles can also be observed in the second row of Fig. 4a.

Through computational fluid dynamics (CFD) modeling, we further analyzed the forces exerting on droplets/particles when passing the gill structures. The flow pattern near gill structure is firstly obtained by using COMSOL Multiphysics (see Methods and Fig. S4). A high pressure area is spotted near the frontal tip of gill structure, as shown in Fig. 4b. The streamline also indicates a vertical velocity of the main flow. When a particle positioning in such a flow, the forces are mainly from the drag, Saffman lift and pressure gradient. We assume the particle or droplet are rigid sphere by neglecting the deformation under the flow. From COMSOL modeling, we are also able to calculate the force exerting on a particle located at position *D* ^+^ . As plotted in Fig. 4b, *F*_y_ is the force value in vertical direction, and *D* ^+^ is the distance ahead of the gill tip. It is found that the droplet size significantly affects the value of *F*_y_. As plotted in Fig. 4b, the larger the droplet is, the larger the force is. *F*_y_ also increases with Re number. More importantly, the distance with a positive *F*_y_ also gets larger, meaning that particles in the flow will have a larger chance to be propelled upward under high Re. We further investigated the influence of gill shape on the vertical force. Different shapes are obtained by rotating the gill tip on a fixed tail (see illustration in Fig. 4c). The angle between the gill tip and tail is defined as α. As seen from the streamlines, the shape completely changes the flow pattern between two adjacent gills even under the same Re = 1.52. When *α* = 220°, strong flow separation occurs behind the gill structure, resulting in a large, captive vortex. In Fig. 4c, we plotted the vertical forces before and behind the tip with different shape angles. With a large *α*, the lift force *F*_y_ ^+^ ahead of the gill tip increases, so does the *F*_y_^-^ behind the tip. The overall effect of *F*_y_ ^+^ and *F*_y_^-^ on particle/droplet flow behavior is evaluated by the deposition probability within a computational domain containing 15 repeated structural elements. When increasing the value of α, the deposition probability first decreases, reaches to its minimum at *α* = 220°, and then increases again. From the streamlines for different α, completely different flow patterns are observed between two adjacent gills even under the same Re = 1.52. When *α* = 220°, strong flow separation occurs behind the gill structure, resulting in a large, captive vortex. The simulation results also show that the overall trend of deposition probability with the increased main flow, which is well aligned with our experimental observation in Fig. 2 and force analysis in Fig. 4b. When the Reynolds number of the main flow exceeds 10, the deposition probability becomes near 0.

### Scalable hybrid filtration device

**Figure 5:**
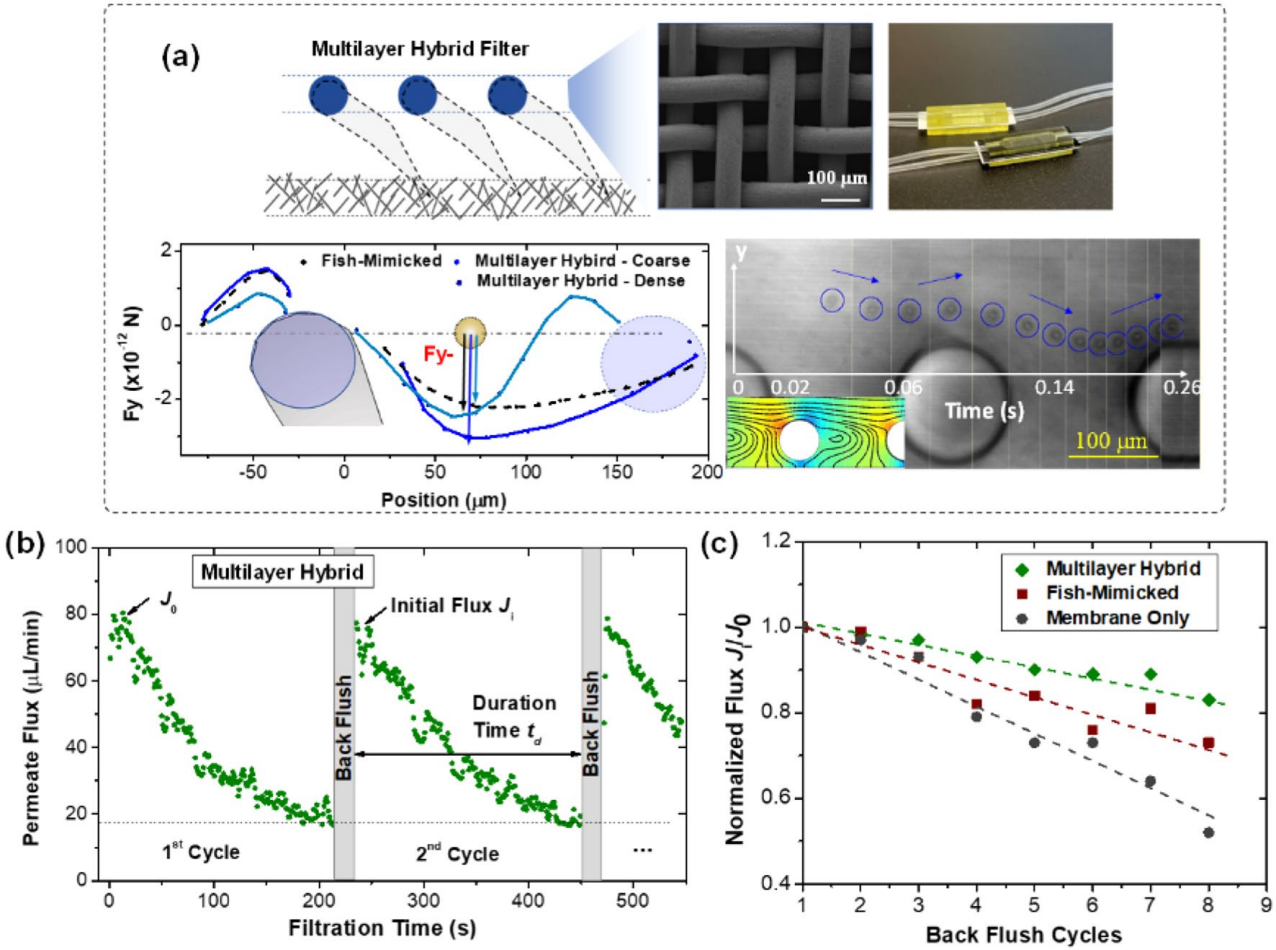
Scalable multilayer hybrid filtration device and flux recovering performance with backflush cleaning. (**a**) Mesh-covered multilayer hybrid filter. The subfigures are mesh surface morphology from SEM images, device outlook from optical images (also see the cross-sectional view of device in Fig. S2b,c), force analysis from simulation results, and ricochet behavior of droplet from flow imaging, respectively. (**b**) Permeate flux change after each backflush cycle of multilayer hybrid filter. (**c**) Comparison of the normalized flux among three different membrane filters.

Noticing that ricochet effect is mainly introduced by the tip of fish gill, we further simplified the gill into a circular shape. In fact, the circular shape structure represents the cross-section of a scalable mesh (see Fig. 5a). The comparison of vertical forces induced by a circular shape and gill shape is provided in Fig. 5a. As expected, the *F*_y_ ^+^ force ahead of the circular structure does not change too much. Although the *F*_y_^-^ shows considerable increase, it can further be controlled by changing the spacing among the wires, namely the mesh pore size. Ricochet of droplet can still be observed above the simplified circular shape from the flow imaging results. To experimentally evaluate the anti-fouling/clogging performance of such a mesh-covered multilayer hybrid filter, we also fabricated a microfluidic filtration device with the proposed printing-on-membrane approach. The fabrication process is similar with fish-mimicked device: right after embedding the membrane in the printed layer, a commercially available copper mesh is then inserted for 3D printing of next layer. The vertical distance between the mesh and membrane can be accurately controlled by the printed layer thickness and layer numbers in between. In this device, we used a copper mesh with thickness of 62 µm, an average pore size of 34 µm, and porosity around 62.6% (see the SEM image in Fig. 5a). The SEM images showing the cross-sectional view of mesh-covered multilayer microfluidic device are given in Fig. S2b,c. Successful fabrication of multilayer hybrid devices demonstrated the great capability of micro-3D printing in additively fabricating new structures on membrane while integrating metallic mesh into an all-in-one device. The easy access to scalable meshes with great varieties in both material and geometry is promising to promote many large-scale industrial implementations.

The as-fabricated membrane filters are examined with backflush cleaning towards practical applications. We characterized the membrane fouling in separating the surfactant-stabilized emulsions followed by the backflush. As a demonstration, the time-dependent declines of permeate flux of the multilayer hybrid filter is plotted in Fig. 5b. The initial permeate flux of a virgin membrane *J*_0_ is around 80 µL/min. When it declines below 20 µL/min, backflush is applied to clean the membrane. Clean water is used in our lab experiments for the backflush (see Fig. S5 of SI for the complete process). In the plotting, the initial permeate flux of each cycle is marked as *J*_i_, and we then compared the normalized initial permeate flux *J*_i_/*J*_0_ among the different aforementioned membrane configurations. As can been seen in Fig. 5c, the normalized flux declined with the increase of filtration cycles for all the configurations, indicating that the hydraulic-irreversible fouling accumulated gradually on membrane surface^45,46^. When an oil droplet gets deposited on membrane surface, it would deform under the high pressure or permeate flux, block membrane pores, or even enter the pores to cause the irreducible fouling. Oil droplet with a larger radius is expected to have a stronger tendency of deformation (see Fig. S6 for oil droplet deformation on membrane surface). From our experimental results in Fig. 5a,c remarkable recovery in the permeate flux was noticed with surface-patterned membrane compared to the bare membrane. Particularly, the FRR of multiplayer hybrid filter is nearly 98% after the 1^st^ cycle of backwash, and still maintains 83% even after 8 cycles. Because the 3D-printed structures and micro-mesh are able to ricochet the large droplets/particles, successfully avoiding membrane contamination. SEM images in Fig. S7 of SI show the surface morphologies of membranes after filtration. Blockage of the pores is observed when using bare membrane, while in the hybrid multilayer filter the membrane is able to main a clean surface with clearly visible morphologies. These results confirmed the outstanding anti-fouling properties of 3D-structured membrane filters. It is worth mentioning that the FRR is highly comparable with or even higher than the values reported in the literature (Fig. 1a), where surface chemistry modification is intensively applied for anti-fouling purpose.

## Conclusions

In this work, we have presented and demonstrated a new kind of 3D-structured membrane filters for sustainable chemical-free water treatment. Inspired by aquatic creatures, we are able to integrate fish-mimicked structures, metallic micromesh and polymeric membrane within single 3D-printed functional filtration device. Their excellent anti-fouling/clogging performance was demonstrated by the high-flux filtration of emulsified oil droplets and plastic micro-particles. The insightful “ricochet” anti-fouling mechanism was also uncovered through in-situ flow observation with microfluidic filters. In this way, we managed to mitigate fouling by micro-3D printed structures and hydrodynamic manipulation, instead of surface chemistry modification with hazardous chemical coatings. We are confident that this approach provides a novel alternative solution to eco-friendly water treatment under the pressing environmental concerns.

In addition, we want to emphasize the versatility and unique advantages of printing-on-membrane, as well as its potential opportunities beyond water treatment applications. 3D printing-on-membrane possesses great design and fabrication flexibility: it can be, homo- or hetero-structured, continuous and/or discontinuous, open and/or closed, mono- or multi-material, and standalone or heterogeneously integrated, single- or multilayer. The vast membrane choices include various materials and different morphologies, from metallic mesh to nanoporous polymeric membranes. By printing 3D structure on membrane, we can also take advantage of increasing choices of printing inks and their composites to build 3D functional structures with desired properties (i.e. elasticity, stiffness, and wettability). In this way, together with the membrane itself, we are able to enable multi-functionalities and heterogeneous physicochemical properties within single assembly-free device.

Potential application fields of printing-on-membrane are equally vast. As one of the most important fields, the use of membranes in microfluidics has become a topic of growing interest. For conventional microfluidic chip fabrication, leakage is a major problem when assembling membrane. Our 3D printing-on-membrane provides an elegant way to overcome this critical problem with self-sealing capability. The chip frame is printed with photo-curable resin and spontaneously bonded with membrane. The benefits of this “print-and-play” membrane device also include the ease of membrane integration, flexibility of chip design, and control/analyzation of mass transport, as demonstrated in this work. Integrated with the enormous variety of materials, morphologies and design options, the microfluidic membrane devices can be readily tailored to other emerging energy, chemistry, bio-engineering, and medical applications.

## Supplementary Information

Supplementary Information 1.

Supplementary Video 1.

Supplementary Video 2.

Supplementary Video 3.

## Data Availability

The datasets used and/or analyzed during the current study available from the corresponding author on reasonable request.
