# Supplementary material for: Biomimetic on-chip filtration enabled by direct micro-3D printing on membrane
Source: Sci Rep. 2022 May 17;12:8178. doi: 10.1038/s41598-022-11738-z (PMC9114119; doi:10.1038/s41598-022-11738-z)
Supplement: Supplementary file 1 — Supplementary Information 1. [file 41598_2022_11738_MOESM1_ESM.pdf]

Supplementary Materials for

Biomimetic On-Chip Filtration enabled by Direct Micro-3D Printing on Membrane

Hongxia Li,[1] *Aikifa Raza,*[1] *Shaojun Yuan,*[2] *Faisal AlMarzooqi,*[3] *Nicholas X. Fang,*[4] *TieJun Zhang*[1*]

[1] Department of Mechanical Engineering, Masdar Institute, Khalifa University of Science and Technology, P.O. Box 127788, Abu Dhabi, UAE.

[2] College of Chemical Engineering, Sichuan University, Chengdu 610065, China.

[3] Department of Chemical Engineering, Masdar Institute, Khalifa University of Science and Technology, P.O. Box 127788, Abu Dhabi, UAE.

[4] Department of Mechanical Engineering, Massachusetts Institute of Technology, 77 Massachusetts Avenue, Cambridge, MA 02139, USA.

[*]Address correspondence to: tiejun.zhang@ku.ac.ae

This PDF file includes:

Table S1
Figs. S1 to S7
Legends for Movies S1 to S3
References S1 to S19

Other Supplementary Materials for this manuscript include the following:

Movies S1 to S3

Table S1: The flux recovery ratio (FRR) for existing anti-fouling/clogging strategies from literature.

	Membrane Type	FRR (%)	Reference
**Bare membranes**	PS-b-P4VP block copolymer	60	[S1]
	Polyvinylidene fluoride hollow fibrous membranes	47	[S2]
	Polyethersulfone ultrafiltration membrane	62	[S3]
	Polypropylene microfiltration membrane	78	[S4]
	Polysulfone membranes	65	[S5]
	Polyvinylidene fluoride ultrafiltration membrane	67	[S6]
	Polyimide membrane	54	[S7]
	Polypropylene membrane	43	[S8]
	Polypropylene macroporous membranes	43	[S9]
	Polyethersulfone ultrafiltration membranes	58	[S10]
**Surface chemistry modification**	Polydopamine/Cysteine surface modified PS-b-P4VP block copolymer	80	[S1]
	Dopamine & quaternary polyethyleneimine coated Polyvinylidene fluoride hollow fiber membrane	94	[S2]
	Polydopamine coated polyethersulfone ultrafiltration membrane	93	[S3]
	Self-polymerized polydopamine followed by hydrolysis of ammonium fluotitanate	90	[S6]
	Methoxypolyethylene glycol grafted polypropylene membrane	86	[S8]
	Amino acids based zwitterionic grafted polyethersulfone ultrafiltration membranes	87	[S10]
	Hydrophilic polyelectrolytes of poly(sulfobetaine methacrylate, poly(sodium p-styrene sulfonate and poly(sodium methacrylate) coated polysulfone membranes	85	[S11]
	Polysulfone/polyethylene glycol hydrophilic membrane	91	[S12]
	Hyperbranched polyglycerol grafted polyethersulfone membranes	95	[S13]
	Poly(2,3-epoxypropyl methacrylate)-functionalized-polyethersulfone [poly(2,3-epoxypropyl methacrylate)f-polyethersulfone]	87	[S14]
**Nano scale modification**	$CuSO_4/H_2O_2$-triggered polydopamine/poly(sulfobetaine methacrylate) coated polypropylene microfiltration membrane	92	[S4]
	Poly(sulfobetaine methacrylate)-functionalized MOF UiO-66-PSBMA coated polysulfone membranes	75	[S5]
	Silver nanoparticles & zwitterionic sulfobetaine methacrylate grafted polyimide membrane	82	[S7]
	Graphene oxide nanosheets on polypropylene macroporous membranes	80	[S9]
	Surface tailored silica nanoparticles grafted polyvinylidene fluoride ultrafiltration membrane	87	[S15]
	Titanium dioxide nanoparticles coated polyvinylidene fluoride membranes	91	[S16]
	Silicon dioxide decorated polypropylene microfiltration membranes	95	[S17]
**Micro-patterning**	Sub-micron scale groove on ultrafiltration polyethersulfone membranes using silica mold	80	[S18]
	Microgrooves on polyamide-imide microfiltration membranes	96	[S19]

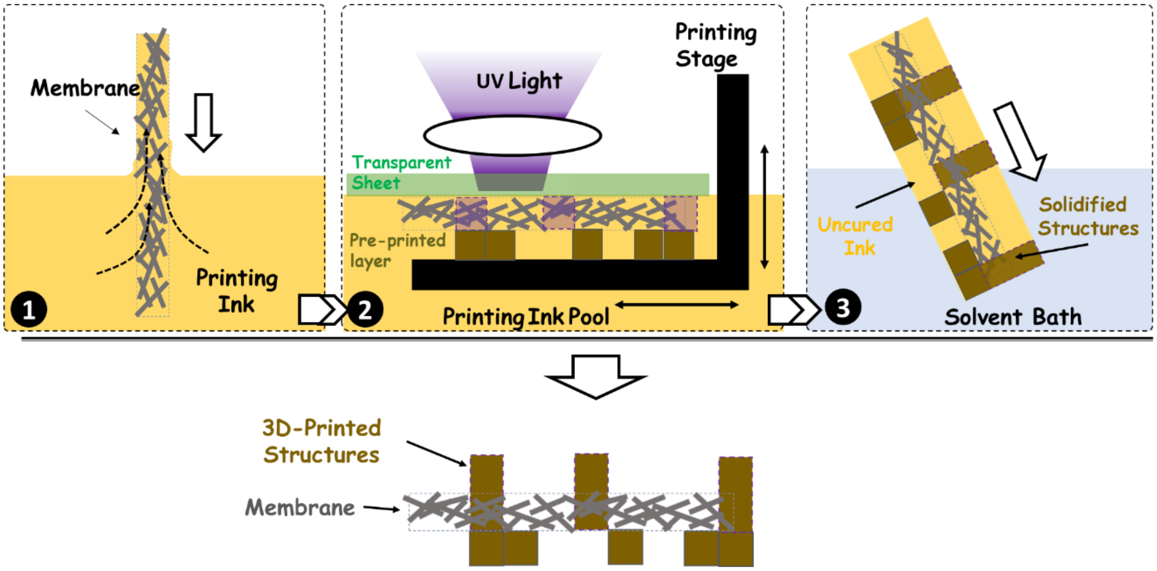

Fig. S1. Schematics showing the detailed printing-on-membrane procedures.

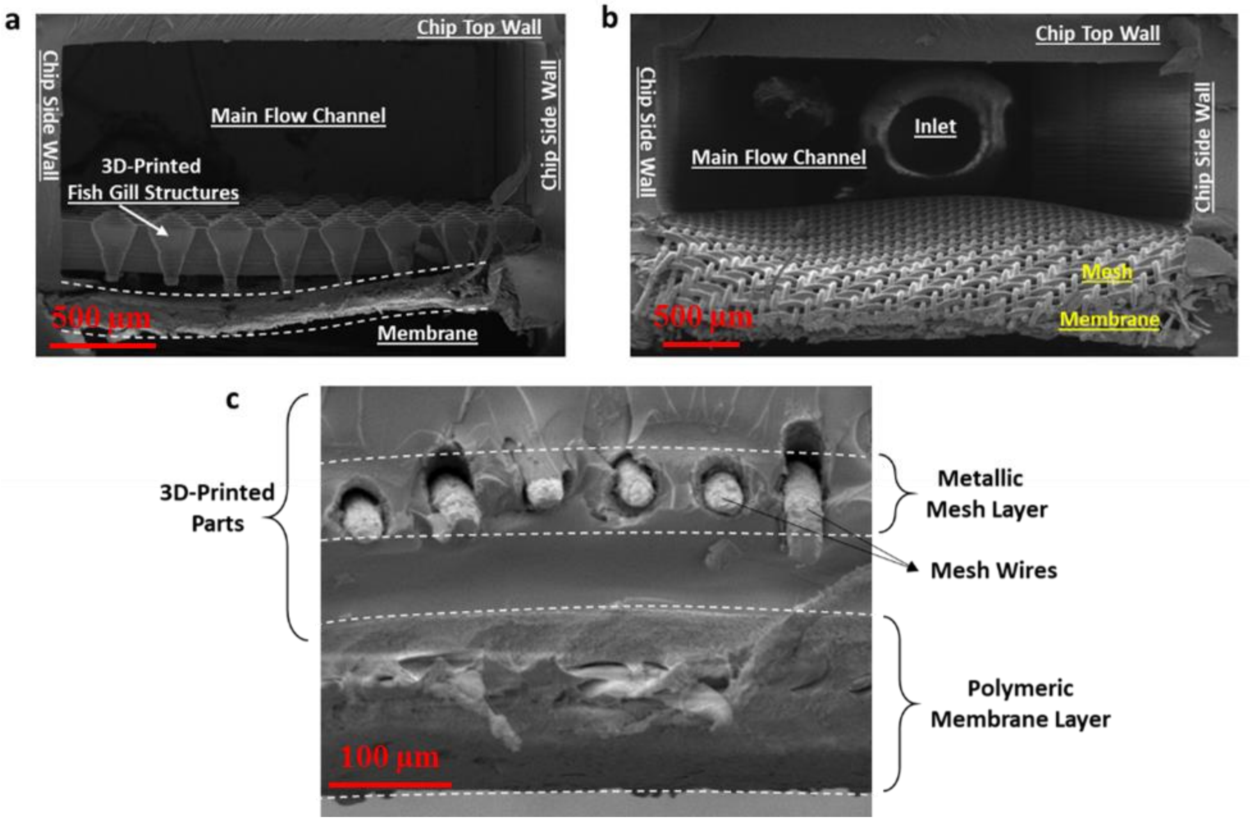

Fig. S2. Cross-sectional SEM images of as-printed on-chip filtration devices. (a) fish-mimicked membrane filtration device and (b) multilayer hybrid membrane/mesh device. (c) High-resolution SEM image of multilayer hybrid device, where the integration of polymeric membrane, metallic mesh and 3D-printed structures. The main flow channel is 1 mm in height and 3.2 mm in width. The thickness of the membrane layer is 140 µm. The diameter of the mesh wire is 34 µm.

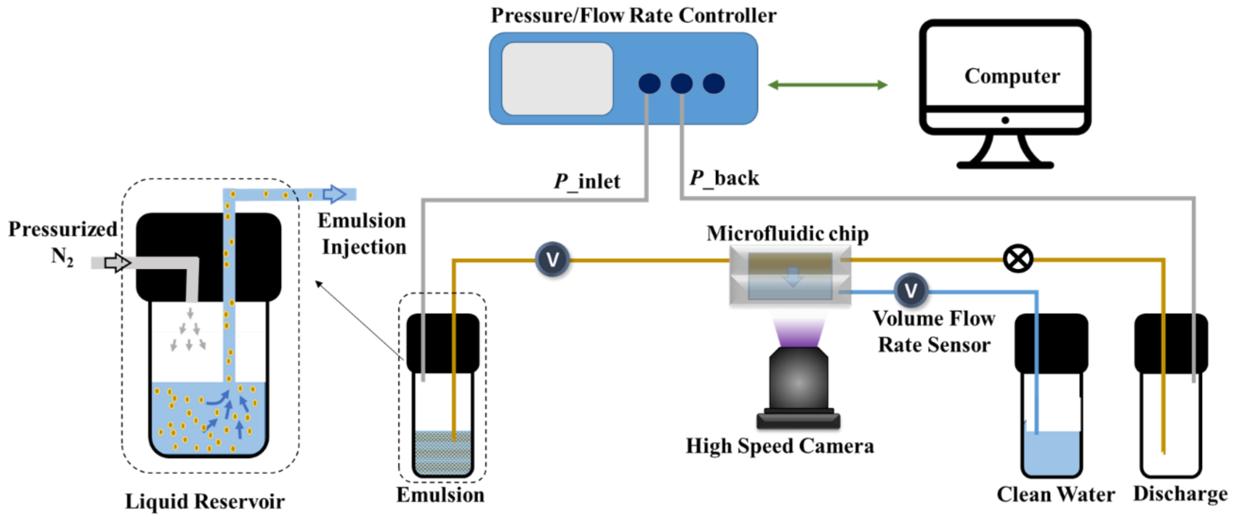

Fig. S3. Schematic diagram of our microfluidic experimental platform integrated with flow controlling and imaging. To conduct the filtration experiments, we have built a microfluidic experimental platform integrated with flow controlling and imaging. The pressure controller is used to regulate the inlet pressure pin during emulsion filtration experiments, so are the backpressure value pout. In this way, we can control the permeate flux crossing the membrane. During the filtration process, the flow rate of the main flow and the permeate are measured by the volumetric flow sensor located at the inlet and the outlet, respectively. In our setup, the microfluidic system is incorporated with a high-speed camera.

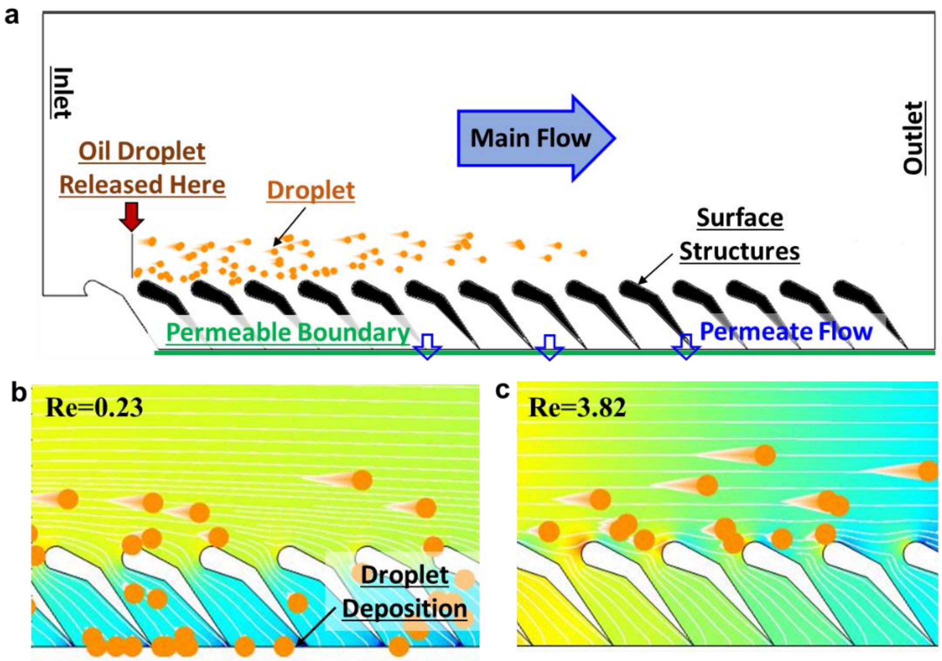

Fig. S4. (a) CFD simulation configurations by using COMSOL Multiphysics. In the simulation, water enters from the left side of the geometry through an inlet with a uniform velocity boundary condition (ranging from 0.01 to 50 mm/s). It then passes over the solid structures (fish gill), where 15 repeated elements are in the calculation domain to avoid any edge effect. The bottom boundary, representing the membrane underneath the gill structures, is set as the uniform permeate velocity of 0.01 mm/s. The outlet at the right side of the calculation domain is set as the outflow boundary condition. (b-c) Oil droplet deposition status on the fish-mimicked membrane surface under two representative Re number. Numerous droplets have been deposited within the view area when Re = 0.23, while no droplet is found when Re increases to 3.82. In the simulation, one hundred oil droplets in total are released within 1 s. After all of the droplets exited from the outlet or deposited on the bottom boundary, the simulation was terminated. The number of droplets get deposited on the bottom boundary is counted as N_d, and the deposition probability is then calculated as $P=N_\mathrm{d}/N_\mathrm{total}$.

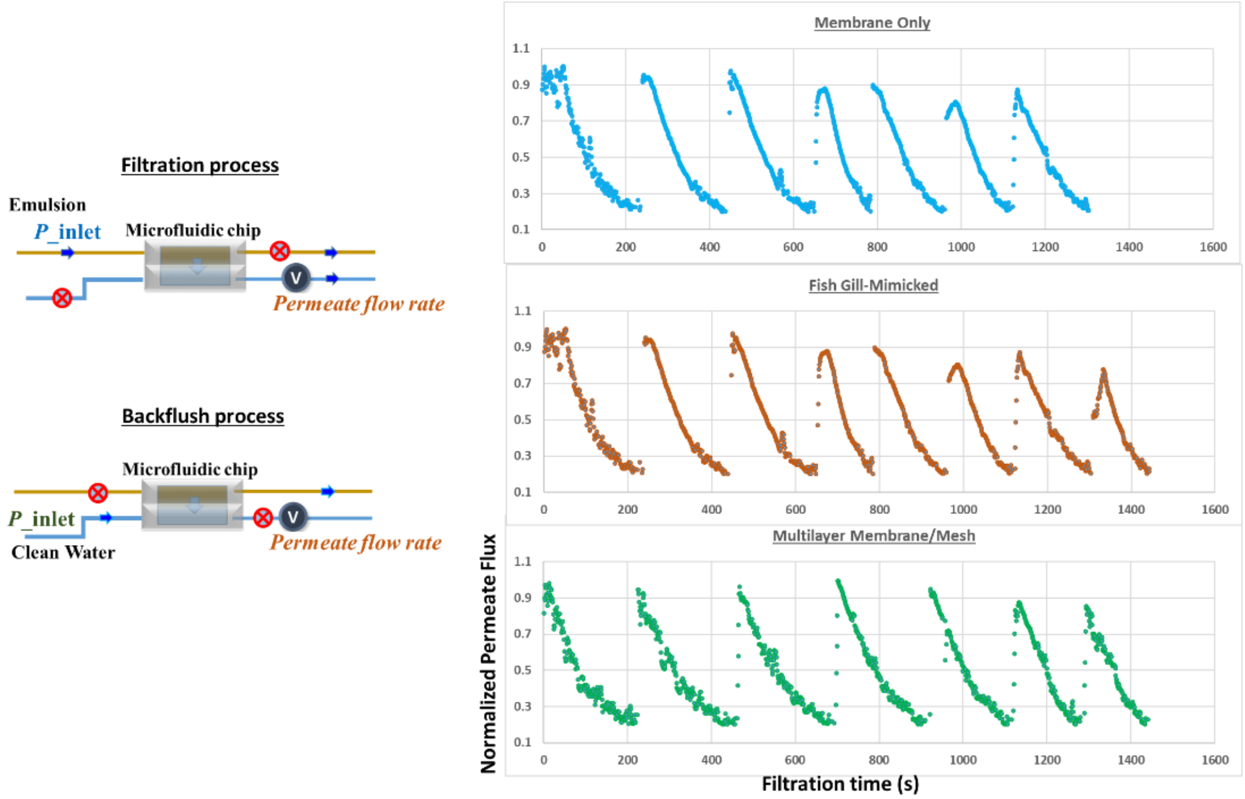

Fig. S5. Filtration performance tests with back flush. Dead-end filtration with periodical backflush was applied to investigate anti-fouling performance of 3D-structured membranes in the emulsion treatment. During the filtration process, the inlet pressure is set as 60 mbar to continuously inject the emulsion into the on-chip membrane filtration device. The permeate flux crossing membrane is monitored by a flow sensor (see the permeate flux profile vs. time at the right side of the figure). When the permeate flux declines to less than 20% of the initial flux, backflush is applied to clean the membrane. During the backflush, clean water is injected from the opposite side of membrane with 120 mbar inlet pressure (see the illustration). The duration time of each back flush is 2 min. The entire filtration tests were conducted for 7 filtration cycles with the membrane-only device, fish-mimicked device, and multilayer hybrid mesh/membrane device, respectively.

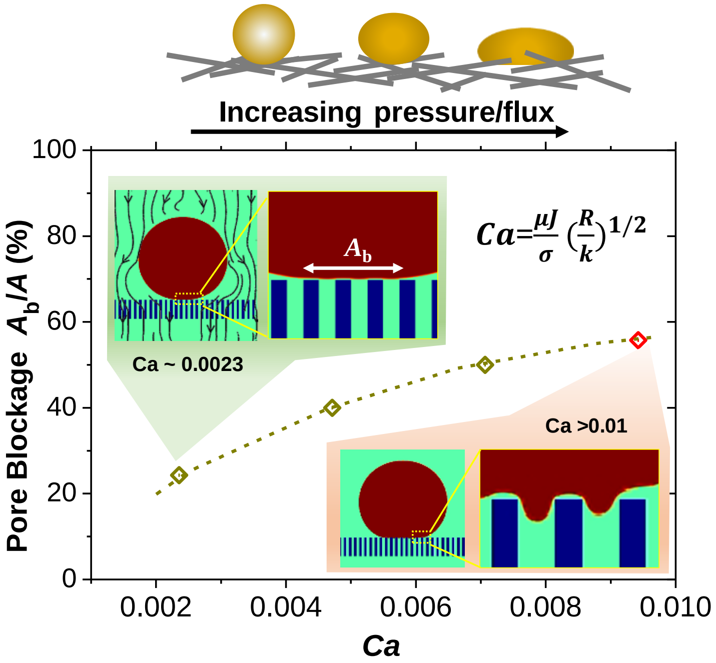

Fig. S6. Droplet deformation and pore blockage area under different Capillary number obtained from lattice Boltzmann simulation. The droplet deformation degree can be characterized by the capillary number, which is a dimensionless parameter representing the ratio of viscous stresses and interfacial tension. For the droplet on a permeate surface, the capillary number is modified as $Ca = \frac{\mu J}{\sigma}(\frac{R}{k})^{1/2}$, where μ and σ are the viscosity of water and interfacial tension of water and oil, respectively, J is the permeate flux (*21*). By using the lattice Boltzmann method, we modeled the deformation of a droplet on simplified membrane with straight water-passing channels. From the first inset image (Ca ~0.0023), we can see the permeate flow and pore blockage by the deformed droplet. The blockage area increases with the increased Ca.

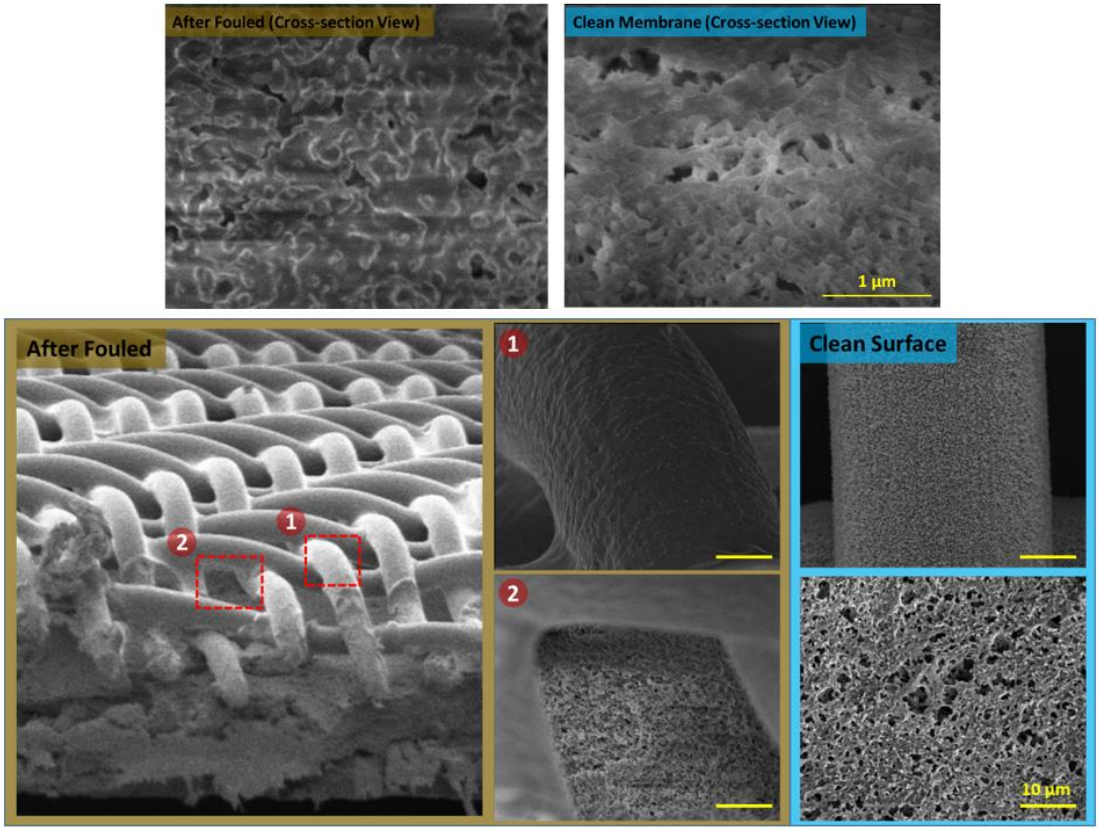

Fig. S7. Morphologies of the membrane surface before and after filtration tests. For the membrane-only device (images in the first row), blockage of the pores is observed after the filtration (the left image) in comparison with the clean membrane (the right image). For the multilayer hybrid mesh/membrane device, we find that oils are inside the surface nanostructures, forming a thin oil layer on mesh wire surfaces after filtration tests (magnified image of red square 1). Thus, it helps membrane underneath the mesh to maintain a clean surface (magnified image of red square 2) from the comparison with the surface morphology of a clean membrane (right-bottom corner image).

Movie Captions

Movie S1. Flow trajectories of oil droplets in fish-mimicked membrane filter captured under the optical microscope.

Movie S2. Flow trajectories of oil droplets in hybrid multilayer filter captured under the optical microscope.

Movie S3. Flow trajectories of plastic micro-particles in fish-mimicked membrane filters captured under the optical microscope.

References:

[S1] R. Shevate, M. Kumar, M. Karunakaran, M. N. Hedhili, and K. V. Peinemann, “Polydopamine/Cysteine surface modified isoporous membranes with self-cleaning properties,” *Journal of Membrane Science*, vol. 529, pp. 185–194, 2017, doi: 10.1016/j.memsci.2017.01.058.

[S2] H. Shi, L. Xue, A. Gao, Y. Fu, Q. Zhou, and L. Zhu, “Fouling-resistant and adhesion-resistant surface modification of dual layer PVDF hollow fiber membrane by dopamine and quaternary polyethyleneimine,” *Journal of Membrane Science*, vol. 498, pp. 39–47, Jan. 2016, doi: 10.1016/j.memsci.2015.09.065.

[S3] Y. Li *et al.*, “Antifouling, high-flux nanofiltration membranes enabled by dual functional polydopamine,” *ACS Applied Materials and Interfaces*, vol. 6, no. 8, pp. 5548–5557, Apr. 2014, doi: 10.1021/am405990g.

[S4] C. Zhang, H. N. Li, Y. Du, M. Q. Ma, and Z. K. Xu, “CuSO4/H2O2-Triggered Polydopamine/Poly(sulfobetaine methacrylate) Coatings for Antifouling Membrane Surfaces,” *Langmuir*, vol. 33, no. 5, pp. 1210–1216, Feb. 2017, doi: 10.1021/acs.langmuir.6b03948.

[S5] H. Sun, B. Tang, and P. Wu, “Development of Hybrid Ultrafiltration Membranes with Improved Water Separation Properties Using Modified Superhydrophilic Metal-Organic Framework Nanoparticles,” *ACS Applied Materials and Interfaces*, vol. 9, no. 25, pp. 21473–21484, Jun. 2017, doi: 10.1021/acsami.7b05504.

[S6] L. Shao, Z. X. Wang, Y. L. Zhang, Z. X. Jiang, and Y. Y. Liu, “A facile strategy to enhance PVDF ultrafiltration membrane performance via self-polymerized polydopamine followed by hydrolysis of ammonium fluotitanate,” *Journal of Membrane Science*, vol. 461, pp. 10–21, Jul. 2014, doi: 10.1016/j.memsci.2014.03.006.

[S7] D. Y. Zhang *et al.*, “Antifouling polyimide membrane with grafted silver nanoparticles and zwitterion,” *Separation and Purification Technology*, vol. 192, pp. 230–239, Feb. 2018, doi: 10.1016/j.seppur.2017.10.018.

[S8] L. L. Wang *et al.*, “Methoxypolyethylene glycol grafting on polypropylene membrane for enhanced antifouling characteristics - Effect of pendant length and grafting density,” *Separation and Purification Technology*, vol. 164, pp. 81–88, May 2016, doi: 10.1016/j.seppur.2016.03.010.

[S9] Z. B. Zhang *et al.*, “Layer-by-layer assembly of graphene oxide on polypropylene macroporous membranes via click chemistry to improve antibacterial and antifouling performance,” *Applied Surface Science*, vol. 332, pp. 300–307, Mar. 2015, doi: 10.1016/j.apsusc.2015.01.193.

[S10] C. Xu *et al.*, “Preparation of PES ultrafiltration membranes with natural amino acids based zwitterionic antifouling surfaces,” *Applied Surface Science*, vol. 385, pp. 130–138, Nov. 2016, doi: 10.1016/j.apsusc.2016.05.084.

[S11] T. Xiang, T. Lu, Y. Xie, W. F. Zhao, S. D. Sun, and C. S. Zhao, “Zwitterionic polymer functionalization of polysulfone membrane with improved antifouling property and blood compatibility by combination of ATRP and click chemistry,” *Acta Biomaterialia*, vol. 40, pp. 162–171, Aug. 2016, doi: 10.1016/j.actbio.2016.03.044.

[S12] N. U. Barambu *et al.*, “Development of polysulfone membrane via vapor-induced phase separation for oil/water emulsion filtration,” *Polymers*, vol. 12, no. 11, pp. 1–16, Nov. 2020, doi: 10.3390/polym12112519.

[S13] X. Li, T. Cai, and T.-S. Chung, "Anti-Fouling Behavior of Hyperbranched Polyglycerol-Grafted Poly(ether sulfone) Hollow Fiber Membranes for Osmotic Power Generation," *Environmental Science & Technology*, vol. 48, no. 16, pp. 9898–9907, Jul. 2014, doi: 10.1021/es5017262.

[S14] N. Misra, V. Kumar, N. K. Goel, and L. Varshney, "Laccase immobilization on radiation synthesized epoxy functionalized polyethersulfone beads and their application for degradation of acid dye," *Polymer*, vol. 55, no. 23, pp. 6017–6024, Nov. 2014, doi: 10.1016/j.polymer.2014.09.035.

[S15] S. Liang, Y. Kang, A. Tiraferri, E. P. Giannelis, X. Huang, and M. Elimelech, "Highly hydrophilic polyvinylidene fluoride (PVDF) ultrafiltration membranes via postfabrication grafting of surface-tailored silica nanoparticles," *ACS Applied Materials and Interfaces*, vol. 5, no. 14, pp. 6694–6703, Jul. 2013, doi: 10.1021/am401462e.

[S16] N. K. O. Cruz, G. U. Semblante, D. B. Senoro, S. J. You, and S. C. Lu, "Dye degradation and antifouling properties of polyvinylidene fluoride/titanium oxide membrane prepared by sol-gel method," *Journal of the Taiwan Institute of Chemical Engineers*, vol. 45, no. 1, pp. 192–201, Jan. 2014, doi: 10.1016/j.jtice.2013.04.011.

[S17] H. C. Yang *et al.*, "Silica-decorated polypropylene microfiltration membranes with a mussel-inspired intermediate layer for oil-in-water emulsion separation," *ACS Applied Materials and Interfaces*, vol. 6, no. 15, pp. 12566–12572, Aug. 2014, doi: 10.1021/am502490j.

[S18] S. H. Maruf *et al.*, "Influence of sub-micron surface patterns on the deposition of model proteins during active filtration," *Journal of Membrane Science*, vol. 444, pp. 420–428, Oct. 2013, doi: 10.1016/j.memsci.2013.05.060.

[S19] A. Asad, M. Rastgar, D. Sameoto, and M. Sadrzadeh, "Gravity assisted super high flux microfiltration polyamide-imide membranes for oil/water emulsion separation," *Journal of Membrane Science*, vol. 621, Mar. 2021, doi: 10.1016/j.memsci.2020.119019.
